# Wear Testing of Moderate Activities of Daily Living Using *In Vivo* Measured Knee Joint Loading

**DOI:** 10.1371/journal.pone.0123155

**Published:** 2015-03-26

**Authors:** Jörn Reinders, Robert Sonntag, Leo Vot, Christian Gibney, Moritz Nowack, Jan Philippe Kretzer

**Affiliations:** Laboratory of Biomechanics and Implant Research, Clinic for Orthopedics and Trauma Surgery, Center for Orthopedics, Trauma Surgery and Spinal Cord Injury, Heidelberg University Hospital, Heidelberg, Germany; Delft University of Technology (TUDelft), NETHERLANDS

## Abstract

Resumption of daily living activities is a basic expectation for patients provided with total knee replacements. However, there is a lack of knowledge regarding the impact of different activities on the wear performance. In this study the wear performance under application of different daily activities has been analyzed. *In vivo* load data for walking, walking downstairs/upstairs, sitting down/standing up, and cycling (50 W & 120 W) has been standardized for wear testing. Wear testing of each activity was carried out on a knee wear simulator. Additionally, ISO walking was tested for reasons of comparison. Wear was assessed gravimetrically and wear particles were analyzed. *In vivo* walking produced the highest overall wear rates, which were determined to be three times higher than ISO walking. Moderate wear rates were determined for walking upstairs and downstairs. Low wear rates were determined for standing up/sitting down and cycling at power levels of 50 W and 120 W. The largest wear particles were observed for cycling. Walking based on *in vivo* data has been shown to be the most wear-relevant activity. Highly demanding activities (stair climbing) produced considerably less wear. Taking into account the expected number of loads, low-impact activities like cycling may have a greater impact on articular wear than highly demanding activities.

## Introduction

The lifetime of artificial knee joints is limited. Wear-associated revisions are known to be a primary reason for long-term failure of total knee replacements (TKR) [1,2]. Wear depends on several factors such as implant-specific factors (e.g. material, design or sterilization technique) [3–5] as well as surgical factors (techniques and surgeon’s skills) with regards to implant alignment and ligament balancing of the TKR [6–8]. Additionally, wear can be related to patient-specific factors (e.g. duration and frequency of gait activities, BMI, muscular conditioning or synovial fluid characteristics) [6,9–11]. The relevance of these factors is still subject to discussion. Nevertheless, the relationship between gait activity and wear seems to be clear. Clinically, it has been shown that increased activity is associated with higher wear [10,12]. The influence of a single activity of daily living (ADL) on the wear performance is, however, not yet known and cannot be assessed clinically.

For in vitro wear testing, walking is considered to be the most frequent [13,14] and internationally standardized (ISO 14243-1) form of loading [15]. Enhanced wear testing with respect to gait activities was intended for harsher conditions that would replicate clinical cases of massive polyethylene (PE) wear and delamination of PE [16–18]. Schwiesau et al. defined testing scenarios of highly demanding activities (walking, stair climbing, squatting) for the reproduction of *in vivo* TKR failure using published literature data on knee joint loading [17,18] and *in vivo* load data of instrumented knee prosthesis [19]. Testing of aged components under highly demanding activities resulted in delamination and significantly increased wear rates compared to testing according to ISO 14243-1 [18]. However, testing was intended to replicate massive PE failure; an isolated investigation of the wear behavior for different activities was not carried out. Muratoglu et al. [16] developed an aggressive wear test of stair climbing and aging of components to evaluate in vitro fatigue wear performance of conventional and cross-linked TKR. Again, fatigue failure (delamination) was successfully replicated for the conventional PE. However, the influence of the activity itself (stair climbing) on the wear behavior remains unknown.

Massive PE wear and delamination are serious forms of clinical wear. Most TKRs, however, remain viable over long periods of use [20,21]. Additionally, failures of TKR due to massive PE wear and delamination seem to have diminished [1,2] due to improvements in PE used in TKR [3]. At the same time, wear-associated aseptic loosening remains one of the most frequent failure modes in long-term assessments of TKR [1,2]. Continuously released submicron wear particles due to articular wear [22,23] might have a greater impact on the survival of TKR rather than the risk of functionality loss because of delamination and massive increased PE wear. Consequently, research on the wear behavior of TKR should not only consider extreme cases of wear and highly demanding activities, but also analyze the effects of moderate loading conditions, which is relevant for the majority of TKR patients.

Resumption of daily living activities and active participation in daily routines are basic expectations of patients provided with TKR [24,25]. Nevertheless, concerns exist regarding the risks of potentially increased wear due to a high activity level of the TKR patient. Knowledge about wear-related risks of different activities is important for the consultant physician and the patient's compliance. Recently, *in vivo* data on forces and torques acting on TKR for different activities have become available (orthoload.com). This data can be used for reliable wear testing of ADL. In this study the wear behavior of the following activities using *in vivo* measured TKR loading is analyzed: walking, walking upstairs, walking downstairs, sitting down and standing up, and cycling with two power levels (50 W & 120 W).

## Materials and Methods

### Determination and definition of test parameters for ADL

For wear testing, kinematics and kinetics of the activities have to be determined. The following parameters are necessary for wear testing: knee flexion/extension angle, axial force, internal-external torque and anterior-posterior forces. These parameters were defined for the following activities: level walking; walking upstairs (stair height 20 cm, without arm support); walking downstairs (stair height 20 cm, without arm support); sitting down and standing up (seat height 45 cm, without arm support); cycling at 60 rpm at a power level of 50 W and 120 W.

For the determination of loading (forces and torques), *in vivo* knee joint loading data available at orthoload.com was used from April 2013. Data of 5 patients (K1L, K2L, K3R, K4R, and K5R) were available for analysis. In terms of cycling data, only one patient (patient K5R) was available.

Loading data of patients has been standardized for two reasons: (1) TKR load data of patients differs between patients (inter-individual) and between different measurements of the same patient (intra-individual); (2) the complex loading profiles have to be simplified (smoothing) to allow for technical realization of simulation. The standardization procedure is described in the next paragraph.

**Fig 1:**
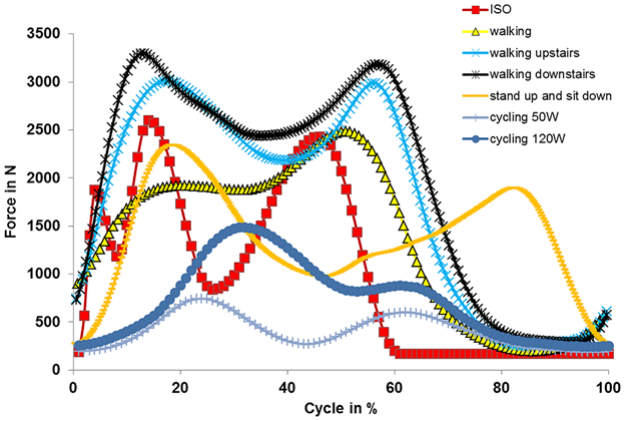
Axial force determined for different activities.

**Fig 2:**
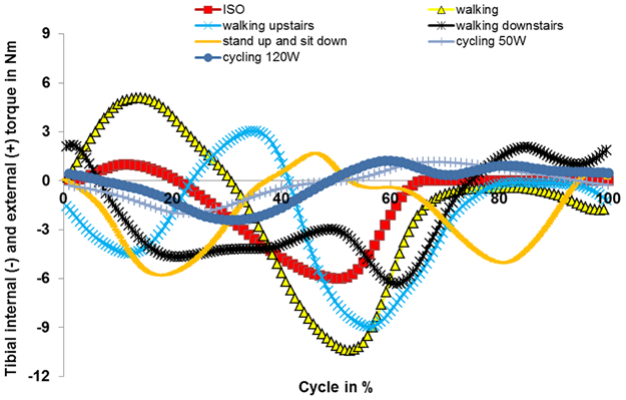
Internal and external torque determined for different activities.

**Fig 3:**
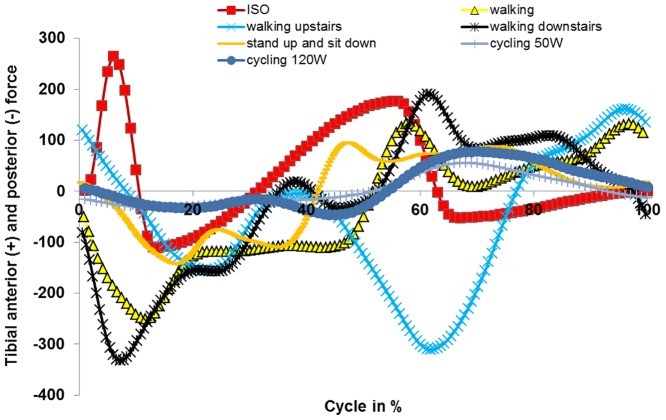
Anterior and posterior force determined for different activities.

The first measured and fully-loaded cycle (after motion initiation) of each activity was used for standardization. Each load cycle for each patient was normalized in time to a cycle representing 100%. Cycles were divided into a load and swing phase. Due to time differences in the execution of movements, the ratio of load and swing phase differed between patients. Thus, an average load and swing phase was defined and the load profile was scaled accordingly (stretched or compressed). For further standardization, the axial profiles were used. Axial profiles were checked for distinctive points (maxima/minima) and their biomechanical setting during activity (e.g. heel strike). After identification of these points, their temporal occurrence was matched between individuals (stretching or compression of load profiles). Profiles of internal-external torques and anterior-posterior forces were adapted in the same way the data points of axial force were previously shifted. Data from all of the patients were then averaged. Profiles were not always consistent between patients (e.g. opposed torques for the same activity) regarding internal-external torque and anterior-posterior forces. To allow for standardization, data from the internal-external torque and anterior-posterior forces were only used from those patients showing agreement in the curve progression of force and torque with the majority of patients. Averaged curve progression for axial force, internal-external torque and anterior-posterior forces are shown in Figs 1–3, respectively. Loading was scaled according to the patient’s weight [26].

**Fig 4:**
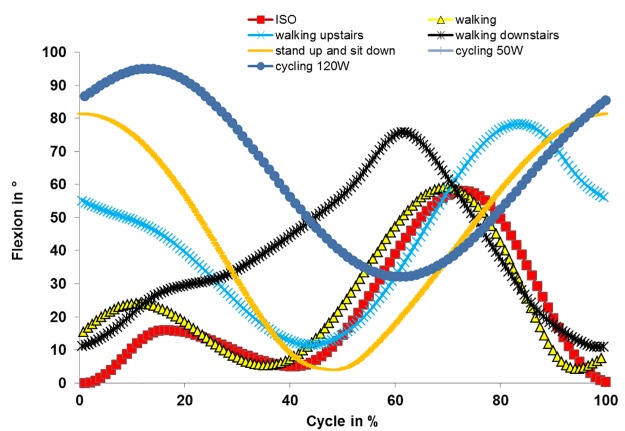
Knee flexion/extension angle determined for different activities.

Knee flexion/extension angle (Fig 4) was determined based on the gait analysis of Rowe et al. [27] for walking, walking upstairs and downstairs, and standing up and sitting down. For cycling, knee flexion/extension angle curve progression was adapted from Ericson et al. [28] and range of motion adapted from Hamai et al. [29].

### Wear testing—General conditions of wear testing

Wear testing was carried out force-controlled on an AMTI knee simulator (Model KS2-6-1000, Advanced Mechanical Technology, Wattertown, MA, USA). The simulator consists of three degrees of freedom (knee flexion-extension angle, internal-external rotation, and anterior-posterior translation) and axial load, which are hydraulically actuated. Force-controlled testing refers to the simulation of the internal-external rotations and anterior-posterior translations. Defined forces and torques are applied to the TKR and kinematics are restrained by soft tissue (ligaments, capsule and soft tissue), alignment (force transmission) and implant design (congruency and friction). The degrees of freedom of medial-lateral translation and varus-valgus rotation are uncoupled and free to move during simulation [30].

Wear tests were run on three articulating wear stations and one axially loaded, soak control station. The soak control was used to measure weight increase that stemmed from the diffusion of solids and liquids of the bovine serum into the PE inlays. PE inserts were presoaked in bovine serum prior to simulation for one year to achieve sufficient saturation of the inserts.

For wear testing, a fixed cruciate-retaining bearing TKR (P.F.C., SIGMA, DePuy Orthopaedics Inc, Warsaw, USA) was used. Tibia-plateaus were aligned with a 7% offset (= 5 mm) of the total plateau width to achieve physiologically higher loading on the medial plateau during simulation [15].

Originally, *in vivo* load data was measured in a cruciate-sacrificing design [26] with a higher degree of coupling compared to the cruciate-retaining design used for wear testing. To allow for consideration of changed restraint characteristics (lower implant restraint and higher ligament restraint) a virtual soft tissue model was used, which simulated the changed restraint characteristics of a cruciate retaining TKR with a sacrificed anterior cruciate ligament and an intact posterior cruciate ligament. This soft tissue model is an mathematical algorithm that calculates the counteracting restraint in real-time, based on the relative anterior-posterior translation/internal-external rotation position during simulation, and reduces the acting load as described by Kretzer et al. [31].

Wear tests were run in a sealed chamber. Bovine serum (PAA Laboratories GmbH, Pasching, Austria) with a protein content of 20 g/L and additives of ethylenediamine tetraacetic acid (7.44 g/L) and sodium acid (1.85 g/L) were used as a substitute for synovial fluid. A testing volume of 250 mL has been used for testing. The lubricating fluid was kept at 37 ± 1°C during simulation.

### Wear testing—procedure of wear testing

First, a wear test with standardized loading according to ISO 14243-1:2009 was carried out. Afterwards wear testing of the six activities with load data derived from instrumented knee prosthesis was carried out. Due to the high number of activities tested (n = 6), the decision was made to carry out testing consecutively on the same implants. Wear testing according to ISO 14243-1:2009 was repeated after termination of the last activity test in order to ascertain the impact of consecutive wear testing on the wear behavior.

Table 1 shows the wear testing procedure. The frequency of execution was determined (orthoload.com) for all activity tests. In a previous study [23], we were able to show that bovine serum degrades time-dependently during simulation with a protective effect on the wear behavior. Using shorter replacement intervals will increase the wear rates, whereas longer time periods of use of the same lubricant fluid will decrease the wear rates. The replacement interval has to be considered when testing is carried out with different test frequencies and, additionally, can be used to shorten the total simulation time. In this study, we used a reference replacement interval (1Hz) of 250,000 cycles and a total simulation period of 2x10^6^ cycles which corresponds to 4 weeks of wear testing. When reducing the test frequency, the replacement interval and the total duration of simulation were adapted accordingly. Each wear test was carried out for a total of four weeks.

**Table 1:** Overview of the order of wear testing, the used frequency, the total test cycles and the point of time of wear measurement and serum replacement.

Test No.	Activity	Frequency in Hz	Total number of test cycles	Serum replacement/wear measurement
1	ISO 14243-1	1	2x10^6^	2.50x10^5^
2	Walking	0.8	1.6x10^6^	2.00x10^5^
3	Walking upstairs	0.6	1.2x10^6^	1.50x10^5^
4	Walking downstairs	0.6	1.2x10^6^	1.50x10^5^
5	Sitting down and standing up	0.3	0.6x10^6^	0.75x10^5^
6	Cycling 50 W	1	2x10^6^	2.50x10^5^
7	Cycling 120 W	1	2x10^6^	2.50x10^5^
8	Recapitulation ISO 14243-1	1	2x10^6^	2.50x10^5^

### Wear analysis

Wear mass was assessed gravimetrically. Therefore, PE components were cleaned and measured according to ISO 14243-2:2009 at each replacement interval. Wear rate was calculated while taking into account the weight increase of the soak control and the density (0.934 mm^3^/mg [32]) of the PE inserts. Wear areas were documented photographically after final test termination.

At the end of each activity test, wear particles were analyzed. Bovine serum was digested through acid digestion according to previously published methods [22,33]. Particles were extracted onto filters with a pore size of 100nm. Wear particles were then analyzed using field emission gun-scanning electron microscopy (FEG-SEM, Leo 1530, Leo, Oberkochen, Germany). A magnification of 25,000x was used for particles less than 0.5 μm and a magnification of 10,000x was used for particles equal or greater than 0.5 μm. Size and morphology of particles were characterized according to ASTM F 1877–05 [34] using a digital image analyzing software (QWin, Leica, Wetzlar, Germany). Mean values for size and morphology were determined based on the number of particles per measured area at both magnifications.

During each test interval the implant kinematics of internal-external rotations and anterior-posterior translations were recorded and analyzed (range of rotation/translation) using the simulator’s own measurement system.

### Statistics

An ANOVA with repeated measures was used to compare differences in wear rates between groups (post hoc test using LSD correction). A p-value of < 0.05 was considered significant. All data is presented with mean ± standard deviation.

## Results

Wear results are presented in Table 2. A wear rate of 6.14 ± 0.46 mm^3^/10^6^ cycles was determined for walking according to ISO 14243-1. A three-fold increase in wear rate of 19.67 ± 1.04 mm^3^/10^6^ cycles (p < 0.01) was determined when *in vivo* walking data was used. All other activities tested resulted in significantly reduced wear rates when compared to *in vivo* walking (p < 0.01 in all cases). Walking upstairs and downstairs resulted in wear rates of 8.88 ± 0.43 mm^3^/10^6^ cycles and of 7.03 ± 0.72 mm^3^/10^6^ cycles, respectively. Sitting down and standing up resulted in a wear rate of 2.91 ± 0.42 mm^3^/10^6^ cycles. The lowest wear rates were observed for cycling. A wear rate of 1.12 ± 0.15 mm^3^/10^6^ cycles was determined for cycling with 50 W and a wear rate 1.19 ± 0.11 mm^3^/10^6^ cycles for cycling at a higher power level of 120 W with no statistical difference between both power levels (p = 0.55). The ISO test was repeated after the last cycling test to check the influence of consecutive wear testing on the wear behavior. An insignificantly increased wear rate compared to the first ISO test of 8.57 ± 2.00 mm^3^/10^6^ cycles was determined (p = 0.15).

**Table 2:** Overview of wear characteristics: wear rates, calculated number of released particles, size and morphology of wear particles.

Activity	Wear rate in mm^3^/10^6^ cycles	Number of particles analyzed	Mean equivalent circle diameter (ECD) in μm	Mean Roundness (R)	Mean Aspect Ratio (AR)
ISO 14243-1	6.14 ± 0.46	1765	0.351	0.572	1.686
Walking	19.67 ± 1.04	1229	0.364	0.528	1.823
Walking upstairs	8.88 ± 0.43	1075	0.359	0.581	1.691
Walking downstairs	7.03 ± 0.72	455	0.380	0.555	1.795
Sitting down and standing up	2.91 ± 0.42	538	0.390	0.591	1.614
Cycling 50 W	1.12 ± 0.15	676	0.423	0.558	1.727
Cycling 120 W	1.19 ± 0.11	543	0.430	0.581	1.654
Recapitulation ISO 14243-1	8.57 ± 2.00	1153	0.386	0.562	1.722

In Table 2, mean wear particle characteristics are shown. Detailed data of both magnifications are shown in S1 Dataset in order to achieve a concise presentation of the data. Generally, submicron sized wear particles were observed in all tests with a mean particle size below 0.5 μm and only small differences in particle morphology (AR, R).

Particle size increased with longer duration of simulation; an increase in particle size from 0.351 μm to 0.386 μm was observed when comparing particles of ISO walking in the first and last testing scenario. However, the largest particles overall with a mean of 0.430 μm were observed for cycling with 120 W and comparable particles with a mean size of 0.423 μm were observed for cycling with 50 W. Smaller particles were observed for walking, walking upstairs/ downstairs and sitting down and standing up.

The mean aspect ratio (Table 2) varied between 1.614 (sitting down and standing up) and 1.823 (*in vivo* walking). Particles with the highest roundness were observed for sitting down and standing up (0.591) and particles showed less roundness (0.528) for *in vivo* walking.

**Fig 5:**
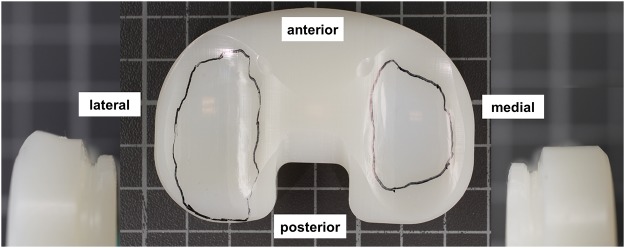
Wear areas on the PE inserts after test termination. In the middle the wear areas are shown. Deformation was visible on the posterior lateral edges of the PE inserts (left); on the medial lateral edges no deformation was visible (right).

Representative wear areas after termination of the last wear test are shown in Fig 5. Larger wear areas were observed on the lateral plateaus and were nearly distributed over the entire plateau area. Smaller wear areas were observed on the medial plateau. Deformation of the PE occurred at the posterior lateral edges of the PE inserts. No pitting or delamination was detected on the surfaces.

Resulting kinematics of internal-external rotation and anterior-posterior translations are shown in Table 3. Highest rotations (17.95 ± 0.60°) and translation (15.96 ± 0.41 mm) were observed for walking upstairs. High kinematics were also observed for walking downstairs (10.96 ± 0.92° and 8.92 ± 1.09 mm). The smallest range of movements was observed for cycling with 50 W and 120 W. Somewhat higher rotations and translations were observed for the simulation of standing up and sitting down. ISO walking produced less rotation but higher translation when compared with *in vivo* walking. However, recapitulation of ISO walking resulted in increased rotations (40%) and translations (12%) compared to the first ISO walking test.

**Table 3:** Kinematics during wear testing.

Activity	Rotation range and distance in °	Max. tibial internal rotation in °	Max. tibial external rotation in °	Translation range and distance in mm	Max. tibial anterior translation in mm	Max. tibial posterior translation in mm
ISO 14243-1	7.96 ± 0.67	7.28 ± 0.36	0.69 ± 0.77	6.81 ± 0.80	3.17 ± 0.21	3.64 ± 0.81
Walking	12.38 ± 0.30	9.26 ± 0.13	3.11 ± 0.21	6.03 ± 0.46	2.77 ± 0.48	3.26 ± 0.05
Walking upstairs	17.95 ± 0.60	17.56 ± 0.59	0.39 ± 0.12	15.96 ± 0.41	10.83 ± 0.44	5.13 ± 0.10
Walking downstairs	10.96 ± 0.92	6.53 ± 0.96	3.80 ± 0.10	8.92 ± 1.09	4.42 ± 0.93	4.50 ± 0.20
Sitting down and standing up	8.73 ± 1.13	7.46 ± 0.84	1.27 ± 0.29	4.49 ± 0.14	1.81 ± 0.26	2.68 ± 0.18
Cycling 50 W	5.27 ± 0.38	4.46 ± 0.14	0.81 ± 0.30	3.12 ± 0.31	-0.07 ± 0.29	3.12 ± 0.31
Cycling 120 W	4.96 ± 0.24	4.04 ± 0.12	0.93 ± 0.19	3.09 ± 0.07	-0.37 ± 0.06	3.46 ± 0.05
Recapitulation ISO 14243-1	11.15 ± 0.31	10.70 ± 0.40	0.45 ± 0.23	7.62 ± 0.04	2.97 ± 0.13	6.26 ± 0.48

## Discussion

In this study *in vivo* load data of instrumented TKR was used to compare the effects of different activities on the PE wear performance. *In vivo* walking was determined to be the activity with the highest impact on wear. All other activities tested resulted in lower wear rates; walking upstairs and downstairs, often termed “highly demanding” activities, resulted in significantly lower wear rates when compared to *in vivo* walking. Cycling with two power levels (50 W and 120 W) resulted in the lowest wear rates overall.

**Fig 6:**
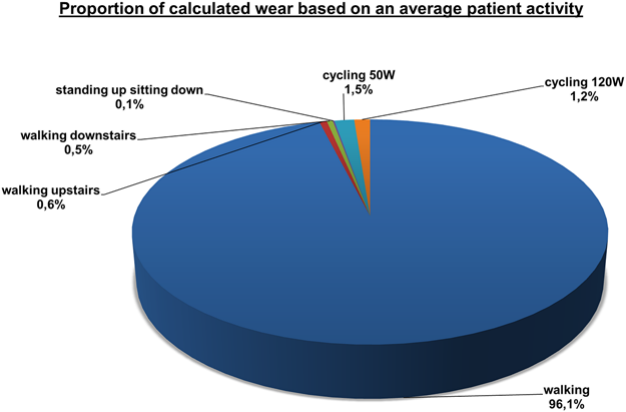
Relative proportion of each activity on the wear behavior calculated for an average activity.

We defined the average patient in order to evaluate the impact of different activities on the *in vivo* wear behavior. It is known that the biological reaction to wear particles is especially related to the size [35] and number/concentration of wear particles [36,37]. In the following consideration we focused on wear rates, as only small differences in wear particle size and morphology were observed in wear particle characteristics for the different activities. Morlock et al. [13] analyzed the duration of frequencies for activities of daily living in THA patients. Using the median number of steps determined for each activity, walking represents 96.6%, walking upstairs and downstairs 2.6% and sitting down and standing up 0.8%, of steps (loads) per day. An average number of 1.1x10^6^ steps per year was determined. Assuming an average patient, this would result in 1.06x10^6^ walking steps per year, 1.43x10^4^ steps for walking upstairs and walking downstairs, respectively and 0.88x10^4^ repetitions of sitting down and standing up. Additionally, cycling can be a frequent everyday activity. Cycling with 50 W or 120 W corresponds to a moderate speed of approximately 15 km/h and 20 km/h, respectively. We assumed a moderate cycling activity of 2500 km/year (average 6.8 km/day). Splitting this into two separate measurements of 1250 km cycling with 15 km/h and 20 km/h would result in 0.23x10^6^ cycles at 120 W and 0.30x10^6^ cycles at 50 W. Taking the determined wear rates into account, the proportion of different activities on the wear behavior can be determined (Fig 6). *In vivo* walking was found to be the predominant activity with the highest wear rates and consequently exhibited the highest impact (96.1%) on calculated wear. Interestingly, cycling with the lowest wear rates has a much higher impact on the calculated wear than “highly demanding” activities like walking upstairs or downstairs. This is related to the fact that cycling is carried out for much longer periods of time and at higher frequencies than walking upstairs and downstairs. Therefore, cycling may be considered to be more influentially related to wear than walking upstairs and downstairs.

Clinically, low-impact sports are commonly recommended after total joint replacement [38]. It is often assumed that high-loading of implants automatically results in higher wear rates. In this study, *in vivo* walking was determined to be the activity with the highest considerable wear rate and is the most frequent activity of those tested. All other tested activities, even with higher loading (walking upstairs/downstairs), showed considerably lower wear rates. Therefore, we believe that recommendations for TKR patients in terms of activities should consider the number of expected loading cycles. As mentioned above, cycling can result in a higher wear burden due to the high number of cycles that are carried out. Highly demanding activities may only have a small effect on wear due to the small number of loading cycles (e.g. golfing or squatting). However, it has to be emphasized that wear is not the only criterion upon which the impact of an activity on *in vivo* performance is evaluated. Highly demanding activities may be more critical with respect to fatigue of the materials as well as fixation (higher interface stress) of the implant than they may be related to wear problems.

The relationship between higher activity and higher wear seems to be clear: higher activity results in more use of the implant and, consequentially, higher wear [10,12]. Taking into account the current concepts of osteolysis and aseptic loosening [36,37], pathological reactions depend on number and concentration of wear particles along with several other parameters. Cell and animal studies indicate that higher particle concentrations result in a higher biological reaction. Thus, higher rates of osteolysis and aseptic loosening should be expected with higher activity. In a recent systematic literature review, the relationship between revision rates, PE wear, and activity reported was analyzed [39]. The authors observed no increase in revision rates due to higher activity despite the fact that higher wear rates were verified for more active patients. Nevertheless, a limitation in most clinical studies is the limited time-period of follow-ups. Aseptic loosening and failure due to PE wear mostly occur in the second decade of use [2]. Therefore, long-term studies with radiological support are necessary to analyze the relationship of wear, activity, and implant failure further. Activity, however, does not only mean wear and automatic implant failure; improvements in general health and bone quality are co-variables of activity that may have a high impact on the outcome of a joint replacement.

Using *in vivo* data of instrumented TKRs for wear testing resulted in unexpected results of wear performance for different activities. On the one hand, the high impact of *in vivo* load data on the walking wear performance compared to ISO walking was surprising. At this point the authors would like to point out that walking according to ISO is also based on *in vivo* walking data but using calculated load data (inverse dynamics). It was also interesting that walking upstairs and downstairs produced comparably small amounts of wear despite exhibiting the highest loads and highest kinematics.

Particularly relevant factors for PE wear generation are known to be 1.) Contact mechanics (load/contact pressure/contact area) [40,41] and 2.) the degree of multidirectional motion [42,43].

1. Three ranks can be distinguished in regards to contact mechanics [41]: at low contact pressures (up to 5.3 MPa), only a low amount of wear can be determined. Increasing contact pressures results in increasing wear rates (up to 10.6 MPa) and further increasing contact pressure results in decreasing wear rates. Higher contact area increases the wear rate when the same contact pressure persists.
2. PE wear depends on the direction of motion. In pin-on-disc studies, unidirectional motion has been shown to result in almost no measureable amount of wear. Multidirectional (cross-shear) results in higher and clinically relevant wear rates. Higher cross-shear (aspect-ratio of wear path) has been shown to be associated with wear rates several times higher [44].

When comparing walking based on ISO standards and *in vivo* data, some relevant differences were observed.

First, differences were found in the curve progression of axial force (Fig 1) despite a comparably maximal axial load. A more continuous high axial load occurred for *in vivo* walking and the unloading in swing phase was reduced and shortened. Calculating the expended axial load (area under curve in Fig 1) results in 1624 Ns for *in vivo* walking compared to 978 Ns for ISO walking, an increase of 66%. Therefore, permanent higher loading occurs when simulating *in vivo* walking. Walking is carried out at relatively low knee flexion/extension angles and, therefore, large contact areas and only moderate contact pressures need to be assumed [45]. Higher axial loading may therefore be related to higher PE wear for *in vivo* walking [40,41]. Additionally, the shortened and reduced unloading during the swing phase may deteriorate the lubrication of the joint and may have a negative impact on wear.

Second, the range of internal-external rotation is increased and the ratio of internal-external rotation to anterior-posterior translation is increased (increased cross-shear). Higher cross-shear has been shown to result in higher wear rates [44].

It may be expected that walking upstairs and downstairs should result in high wear rates due to it having the highest loading of all activities and highest resulting kinematics. The most obvious difference to *in vivo* walking with the highest wear rate is the changed knee flexion/extension angle. Axial loading occurs at a considerably higher knee flexion/extension angle compared to walking. This may result in smaller contact areas [45] and higher contact pressure with a wear-reducing effect [41]. High secondary motions (internal-external rotations and anterior-posterior translations) were observed that are known to be linked to higher wear [44]. However, even for cross-shear effects and its relationship to wear, it has to be considered that the wear-increasing effects are related to the contact area. A smaller contact area results in smaller effects of cross-shear on wear [46]. Regarding material fatigue and durability of the implant, the high loading may be considered to be critically influential. A similar trend is observed for edge loading in PE-based total hip bearings; edge loading is of concern regarding fatigue of the PE. However, accelerated wear due to edge loading does not occur [47].

There are several limitations of this study which have to be mentioned. Standardization of *in vivo* loading resulted in simplified loading profiles. *In vivo* loading actuated by muscles is more sophisticated than can be technically reproduced. This may have an impact on the wear behavior. In a recent publication, Bergmann et al. [48] published standardized loadings and kinematics (knee flexion/extension angle) for several activities. This data was not available when wear testing was carried out. There was, however, a high degree of agreement in the curve progression of forces and torques in our study, albeit with a lower loading. Based on Bergmann et al. [48], the data used in this wear test study corresponds to an average 75 kg patient and an even higher patient weight was used for calculation in this study [26]. A limitation of wear testing for cycling is that the load data is based on only one patient. Regarding used knee flexion/extension angle in this study it should be mentioned that gait analysis data and used load data are derived from different patient collective.

Standardized, prolonged testing may result in a bias of wear results. It is known that PE molecules can orientate in the direction of loading which strengthens the material and decreases wear [42]. More challenging activities like walking upstairs differ more in their execution of motion than less technically challenging activities like cycling (controlled by pedals). This may result in differing kinematics in the execution of the same activity with an increasing wear effect for more technically demanding activities.

Consecutive wear testing is a limitation of this study. A non-significant increased wear rate was observed in the recapitulation of the ISO walking test. We attribute the increased wear rate in the recapitulation test to higher kinematics resulting from loss of congruency (creep) and decreased roughness of the PE surface (burnishing). However, none of the tested activities showed an increased wear behavior when compared to *in vivo* walking. Therefore, the qualitative results of this study are not biased by the consecutive use of implants.

The activities tested were carried out with moderate loading. Future studies may use maximal loads for wear testing for each activity. Additionally, load data (orthoload.com) can be used to analyze the effects of individual loading and to analyze the individual wear characteristics of different patients.

## Conclusion

Walking seems to be the most relevant activity when investigating wear effects. Highly demanding activities like climbing have been shown to have only a moderate impact on wear. Low-impact sports (cycling) have been shown to generate the lowest wear rate, but the expected high number of loads may be an exacerbating factor in the evaluation of wear performance.

## Supporting Information

S1 Dataset Details of wear particle analysis at both analyzed magnifications. (DOCX)
