# Supplementary material for: Wear Testing of Moderate Activities of Daily Living Using In Vivo Measured Knee Joint Loading
Source: PLoS One. 2015 Mar 26;10(3):e0123155. doi: 10.1371/journal.pone.0123155 (PMC4374780; doi:10.1371/journal.pone.0123155)
Supplement: S1 Dataset — (DOCX) [file pone.0123155.s001.docx]

Supporting information S1

| Activity | Magnification | Number of particles analyzed | Relative fraction (based on particles per measured area) | Mean Equivalent circle diameter (ECD) in µm | Equivalent circle diameter (ECD) in µm | Mean Roundness (R) | Roundness (R) | Mean Aspect Ratio (AR) | Aspect Ratio (AR) |
| --- | --- | --- | --- | --- | --- | --- | --- | --- | --- |
| ISO 14243-1 | 10,000 | 937 | 15.2 % | 0.351 | 0.75 ± 0.30 | 0.572 | 0.50 ± 0.14 | 1.686 | 1.82 ± 0.53 |
|  | 25,000 | 828 | 84.8 % |  | 0.28 ± 0.08 |  | 0.59 ± 0.13 |  | 1.66 ± 0.40 |
| Walking | 10,000 | 711 | 17.6 % | 0.364 | 0.71 ± 0.28 | 0.528 | 0.43 ± 0.15 | 1.823 | 2.03 ± 0.68 |
|  | 25,000 | 518 | 82.4 % |  | 0.29 ± 0.08 |  | 0.55 ± 0.14 |  | 1.79 ± 0.51 |
| Walking upstairs | 10,000 | 615 | 16.1 % | 0.359 | 0.74 ± 0.26 | 0.581 | 0.50 ± 0.15 | 1.691 | 1.83 ± 0.55 |
|  | 25,000 | 460 | 83.9 % |  | 0.29 ± 0.08 |  | 0.60 ± 0.14 |  | 1.66 ± 0.44 |
| Walking downstairs | 10,000 | 282 | 19.7 % | 0.380 | 0.74 ± 0.27 | 0.555 | 0.46 ± 0.17 | 1.795 | 2.04 ± 0.79 |
|  | 25,000 | 173 | 80.3 % |  | 0.29 ± 0.09 |  | 0.58 ± 0.13 |  | 1.74 ± 0.66 |
| Sitting down and standing up | 10,000 | 358 | 21.9 % | 0.390 | 0.82 ± 0.36 | 0.591 | 0.54 ± 0.14 | 1.614 | 1.71 ± 0.49 |
|  | 25,000 | 180 | 78.1 % |  | 0.27 ± 0.08 |  | 0.60 ± 0.12 |  | 1.59 ± 0.29 |
| Cycling 50W | 10,000 | 453 | 24.0 % | 0.423 | 0.74 ± 0.29 | 0.558 | 0.52 ± 0.14 | 1.727 | 1.79 ± 0.55 |
|  | 25,000 | 223 | 76.0 % |  | 0.32 ± 0.08 |  | 0.57 ± 0.14 |  | 1.71 ± 0.46 |
| Cycling 120W | 10,000 | 385 | 27.5 % | 0.430 | 0.77 ± 0.30 | 0.581 | 0.53 ± 0.14 | 1.654 | 1.76 ± 0.60 |
|  | 25,000 | 158 | 72.5 % |  | 0.30 ± 0.08 |  | 0.60 ± 0.12 |  | 1.61 ± 0.35 |
| Recapitulation ISO force-controlled | 10,000 | 724 | 20.8 % | 0.386 | 0.71 ± 0.25 | 0.562 | 0.50 ± 0.15 | 1.722 | 1.86 ± 0.71 |
|  | 25,000 | 429 | 79.2 % |  | 0.30 ± 0.08 |  | 0.58 ± 0.13 |  | 1.69 ± 0.40 |
